# Longitudinal Association Between Education and Disability in Older Adults Living in Iceland

**DOI:** 10.1093/geroni/igaa057.1274

**Published:** 2020-12-16

**Authors:** Milan Chang, Olof Geirsdottir, Lenore Launer, Vilmundur Gudnasson, Palmi Jonsson, Alfons Ramel, Milan Chang Gudjonsson

**Affiliations:** 1 University of Iceland, Reykjavik, Iceland; 2 National Institute on Aging, Bethesda, Maryland, United States; 3 University of Iceland, Reykjavik, Capital area, Iceland

## Abstract

BACKGROUND: Disabilities among older adults are associated with cumulative adversities such as low socioeconomic status (SES), poor nutrition, and lack of access to medical care and education. However, there is little evidence on the long-term association between education and disability status among older adults in Iceland. The aim of the study was to examine the association between mid-life education and prevalence of disability in activities of daily living (ADL) and mobility disability in late-life using 25 years of longitudinal data. METHODS: A large community-based population residing in Reykjavik, Iceland participated in a longitudinal study with an average of 25 years of follow-up (N=5764, mean age 77±6 yrs, 57.7% of women) Mid-life education was categorized into 2 groups (primary and secondary versus college and university). Disability status in late life was defined with ADL and mobility disability with a binary outcome (no difficulty versus any difficulty). Logistic regression analysis was used to examine the association. RESULTS: After controlling for age and gender, and midlife health risk factors, those who had high education at mid-life were less likely to have ADL disability (Odds Ratio (OR) = 0.75, 95% Confidence Interval (CI): 0.64 ~ 0.88, P ≤ 0.001) and mobility disability (OR = 0.72, 95% CI: 0.61 ~ 0.86, P < 0.001) compared with those who had low education in mid-life. CONCLUSION: People with high mid-life education were less likely to have ADL and mobility disability after 25 years later.

